# Relationships of Sources of Meaning and Resilience With Meaningfulness and Satisfaction With Life: A Population-Based Study of Norwegians in Late Adulthood

**DOI:** 10.3389/fpsyg.2021.685125

**Published:** 2021-12-02

**Authors:** Torgeir Sørensen, Knut Hestad, Ellen Karine Grov

**Affiliations:** ^1^Faculty of Health Studies, VID Specialized University, Sandnes, Norway; ^2^Center for Psychology of Religion, Innlandet Hospital Trust, Hamar, Norway; ^3^Inland Norway University of Applied Sciences, Elverum, Norway; ^4^Faculty of Health Sciences, Oslo Metropolitan University, Oslo, Norway

**Keywords:** meaning, meaningfulness, sources of meaning, satisfaction with life, resilience, old adults

## Abstract

Health-promoting initiatives incorporating meaning-making to enhance the well-being of people in late adulthood are important, particularly as the number of older people is increasing. Resilience and sources of meaning may be related to individuals’ experience of meaningfulness and satisfaction with life. However, few studies have investigated these relations among people in late adulthood. In the present exploratory study, we asked the following questions: What are the differences regarding scores on sources of meaning, resilience, meaningfulness, and satisfaction between people in late adulthood (≥65) and other adults (18–64)? What is the association between sources of meaning and meaningfulness, and between resilience and meaningfulness? What is the association between sources of meaning and satisfaction with life, and between resilience and satisfaction with life? A cross-sectional design was used. A population-based sample of 925 participants (aged 18–91 years) was recruited from the National Population Register in Norway. Of these, 219 participants were 65 years old and older (mean age 73 years). Additionally, sub-analyses for the age-group ≥ 75 (*N* = 71) were performed. Independent-samples *t*-tests, chi-square tests, one-way ANOVA, and linear regressions adjusted for demographics, anxiety, and depression were performed utilizing standardized questionnaires. It was found that people in late adulthood (≥65 years) scored significantly higher on meaningfulness compared to younger adults (18–64). Of the sources of meaning, vertical self-transcendence, including explicit religiosity and spirituality, had the strongest relation to meaningfulness for people in late adulthood, after adjusting for demographics, anxiety, and depression. For the same group, accomplishment, including generativity and unselfish engagement with the surroundings and future generations, also stood out as a prominent source of meaning when related to meaningfulness. No sources of meaning were associated with satisfaction with life in the older group. No associations between resilience and meaningfulness, nor between resilience and satisfaction with life, were found among people in late adulthood. However, positive associations were found between resilience and meaningfulness, as well as between resilience and satisfaction with life, in the 18–64 age group. Longitudinal research and interventional studies are needed to confirm whether the designated sources contribute to meaningfulness in a Norwegian context. The implications of the findings are discussed.

## Introduction

Longevity is increasing in most countries. Among people in late adulthood, many face multiple health conditions and mobility problems, and relatively high rates of chronic diseases and disability are observed among older people (≥65 years) (Eurostat, 2020, p. 50). At the same time, challenges related to changes in their life situations, such as loss of spouse and retirement, are emphasized (Norwegian Government, 2017). A goal of Norwegian authorities is to facilitate older people staying at home as long as possible due to the positive impact of dwelling in known surroundings (Norwegian Government, 2017). It is recognized that several approaches are needed to support older people when living at home. Among these, health-promoting initiatives that enhance meaningfulness and well-being have been suggested (Knitzek et al., 2021). Meaning-making and maintenance of meaning have long been considered a key aspect of successful adaption to aging (Reker and Wong, 2012), since discovery and creation of meaning through inner and spiritual resources may be a way of transcending for instance personal losses and despair in old age (Wong, 1989). Still, health promotion through meaning-making and other inherent resources like resilience may be underestimated assets for people’s well-being, at least in Norway and the Scandinavian countries (Knitzek et al., 2021). A contextual investigation of older adults’ experience of meaningfulness and satisfaction with life is needed to inform policy development and the service implementation of health promotion initiatives and to better understand the relationship of these factors to sources of meaning and resilience. As a first step, the present study involves an exploratory investigation of this issue in Norway.

According to Schnell (2021), *meaningfulness* is the basic trust that life is worth living, based on an appraisal of life as coherent, significant, oriented, and belonging. Coherence is linked to life making sense, orientation is about having goals and aims in life, while significance emphasizes life’s inherent values (Martela and Steger, 2016). Previous research has found meaningfulness to be associated with more hopefulness and optimism (Damásio et al., 2013) and higher levels of self-determination and social integration (Kashdan and Breen, 2007). Meanwhile, meaningfulness is negatively correlated with anxiety, depression, and post-traumatic stress (Sørensen et al., 2019; Schnell, 2021). Meaningfulness is found to be higher among those at later life stages (Steger et al., 2006). Meaningfulness is also found to positively impact health-related outcomes of people in late adulthood, helping them to sustain a healthy life and contributing to a reduced risk of premature mortality (Steptoe and Fancourt, 2019).

Meaningfulness is linked to individuals’ engagement in a variety of orientations represented by *sources of meaning*, depending on which purpose they perceive as significant (Schnell, 2021). Sources of meaning have been identified by several research programs (Wong, 1998; Reker, 2000; Bar-Tur et al., 2001; Schnell, 2009, 2011) and form the grounds of the meaning experience. The 26 sources identified by Schnell (2009, 2011) appear in the six dimensions of the Norwegian validation of the Sources of Meaning and Meaning in Life Questionnaire (SoMe) (Sørensen et al., 2019). These six dimensions are as follows: (1) Well-being and relatedness pertain to commitment to enjoyment and sensitivity in private and with company; (2) Order and tradition pertain to commitment to principles, common sense, and the tried and tested; (3) Vertical self-transcendence pertains to a search for an immaterial supernatural reality through explicit religiosity and spirituality, while (4) Horizontal self-transcendence pertains to a commitment to worldly affairs beyond immediate concerns; (5) Accomplishment pertains to achievement, development, power, challenge, knowledge, and generativity; and (6) Liberality pertains to freedom, individualism, comfort, and creativity (Schnell, 2009, 2011; Sørensen et al., 2019).

It has been found that a deeper sense of meaning and higher levels of satisfaction with life are achieved through self-transcendence and collectivism compared to occupation with sources related to self-preoccupation and realization of personal potential, as the latter may be more related to situational meaning where meaning-making appears in everyday life experiences (Reker and Wong, 2012). For older adults, such a pattern could be explained by Törnstam’s (1989) gerotranscendence theory, emphasizing a shift of meta-perspective from a materialistic and rational view toward a more value-based, communal and transcendent focus in older age. This claim finds support in previous research on older adults, where communal and transcendental sources seem to be more prominent than the individualistic ones (Bar-Tur et al., 2001; Schnell, 2009).

*Satisfaction with life* is a distinct construct representing a cognitive and global evaluation of the quality of one’s life as a whole in view of beliefs and expectations (Keyes et al., 2002; Pavot and Diener, 2008). It may be a paradox that satisfaction with life has been found to be higher among older adults while health challenges and other limitations are increasing in this life phase (Gana et al., 2013). This might be explained by the fact that older people optimize their performance in selected and well-known domains and values, compensating for their inevitable limitations (Riediger et al., 2005).

Differentiation can be seen regarding various sources related to meaningfulness and satisfaction with life, respectively. In the literature, these differences have been explained by meaningfulness representing eudaimonic well-being, focusing on personal growth and striving for meaning (Huta, 2018), whereas satisfaction with life is characterized as hedonic, focusing on basic needs and desires in the here and now (Baumeister et al., 2013). Such a differentiation is shown in a study of older adults (≥65 years) in residential care, where the sources personal growth, spirituality/religion, and interpersonal relationships were positively related to personal meaning, while family and leisure were positively related to satisfaction with life (Dewitte et al., 2021). A study of active seniors found no relationship between eudaimonic sources of meaning and life satisfaction (Penick and Fallshore, 2005). Thus, it was suggested that personal meaning may not serve as a major source of life satisfaction.

*Resilience* may be defined as the personal qualities that enable individuals to thrive, and it is also viewed as an expression of successful stress-coping ability (Connor and Davidson, 2003). When facing stressors, threats, or demanding life situations, resilience represents the dynamic process of positive adaption and the ability to maintain or regain mental health, whereby individuals reintegrate in life despite experiencing adversity (Herman et al., 2011). Resilience may vary with age and is proposed to be lower in older than in younger people (Rothermund and Brandtstädter, 2003).

A qualitative interpretive meta-synthesis (Bolton et al., 2016) pointed at several partially coincident constructs between resilience and meaningfulness. The most prominent factors were external connections emphasizing relations and social support; meaningfulness highlighting existential aloneness, meaning-making, and spiritual/religious practices; in addition to a positive perspective on life. In a population sample, resilience was positively correlated with meaningfulness (Sørensen et al., 2019). It should be mentioned, though, that the direction between the factors in the two latter mentioned studies could not be determined.

When investigating the relationship between resilience and satisfaction with life, it has been shown that resilience may increase satisfaction with life among patients with spinal cord injuries and their family members (Jones et al., 2019). In another selected sample, there was a positive association between resilience and satisfaction with life, at the same time as a negative association between resilience and depression was found among police officers working during a natural disaster (McCanlies et al., 2018). As an emotional strength or resource, resilience may serve as a protective health factor in aging. For instance, in a sample of adults (30–80 years), a significant inverse association between resilience and depression was observed for persons over 70 years (Leppert and Strauss, 2011). On the other hand, population-based investigations on this issue are scarce, especially regarding people in late adulthood.

From an overall perspective, it has been suggested that personal meaning and its sources can be viewed as potentially significant components of psychological resilience (Reker and Wong, 2012), contributing to greater hardiness when facing demanding life situations. On the other hand, greater hardiness could also contribute to a more meaningful life. However, research has largely failed to include the meaning constructs in conceptualizations and measures of resilience and vice versa. A supplement of the picture may be seen through the lens of gerotranscendence theory (Törnstam, 1989), according to which people in old age emphasize communal and transcendental views and sources, which may positively impact meaningfulness and satisfaction with life to a larger extent than rational and materialistic concerns (Törnstam, 1997). Several factors may be of significance when facing age-related challenges. The targeted factors in the present study, sources of meaning, resilience, meaningfulness, and satisfaction with life, may function as resistance against stressful events, adverse outcomes of the adaption process, negative health outcomes, and the development of disorders (Davydow et al., 2010). Since the study is the first of its kind in the Norwegian context, the approach was exploratory. The study is cross-sectional and aims with its exploratory design to present an openness toward directions between the dependent and independent variables, respectively.

In the present study, we aimed to investigate the presented concepts and how they emerge among people in late adulthood (≥65) as well as the associations between the concepts in a Norwegian context. A comparison with the rest of the population (18–64) is done to determine what is important for those in old age. New insights on these matters may generate knowledge crucial for policy and service development on health promotion, supporting people in late adulthood to stay at home as long as possible.

Against this background, and with an emphasis on people in late adulthood, we present the following research questions in this exploratory study: (1) What are the differences regarding scores on sources of meaning, resilience, meaningfulness, and satisfaction with life between people in late adulthood (≥65) and other adults (18–64)? (2) What is the association between sources of meaning and meaningfulness, and between resilience and meaningfulness? (3) What is the association between sources of meaning and satisfaction with life, and between resilience and satisfaction with life?

## Materials and Methods

### Design and Sample

The present study used a cross-sectional research design. A population-based sample was recruited through a randomized draw from the Norwegian National Registry. A total of 7,500 people 18 years old and older were invited via an invitation letter that was sent by postal mail. Of those invited, 790 participants responded to the questionnaire, which was returned in a prepaid envelope, while 175 respondents completed the questionnaire online via Checkbox. Of the 965 respondents, 22 were omitted due to missing values of 5% or more. In addition, 18 cases were excluded due to identified multivariate outliers. Consequently, the final total sample consisted of 925 respondents, of which 54.5% were women and 45.5% were men (49.4 and 50.6% among those invited, respectively). The age range was 18–91, and the mean age was 51.7 years (SD = 16.6; the mean age was 47.7 among those invited). Most of the participants were in pair-bonded relationships (70.3%), and 59.6% had more than 12 years of education. Of the total sample, 219 persons were aged 65 years or older.

### Measures

#### Demographic Variables

Socio-demographics were measured by gender, age, education level as an expression of socio-economic status (≤12 years, >12 years), and civil status (single, pair-bonded).

#### Meaningfulness and Sources of Meaning

We used the Sources of Meaning and Meaning in Life Questionnaire (SoMe) (Schnell, 2009, 2011) to measure “Meaningfulness” (5 items, e.g., “I lead a fulfilled life”) (α = 0.71) as well as to measure sources of meaning through 26 subscales with a total of 141 items. All items in the SoMe are rated on a six-point Likert scale (0 = totally disagree; 5 = totally agree). In a validation of the SoMe in the Norwegian context, the 26 subscales were grouped into six dimensions (Sørensen et al., 2019). Mean sum scores were calculated for the subscales and the six dimensions, ranging from 0 to 5. Cronbach’s alpha’s for the six dimensions were “Well-being and relatedness” (27 items, e.g., “I often think about how I can please others,” α = 0.82), “Order and tradition” (24 items, e.g., “I like to stick to habits,” α = 0.81), “Vertical self-transcendence” (8 items, e.g., “I draw strength from my faith,” α = 0.77), “Horizontal self-transcendence” (28 items, e.g., “I place great emphasis on living in harmony with myself and others,” α = 0.73), “Accomplishment” (31 items, e.g., “I strive to do something for the generations after me,” α = 0.84), and “Liberality” (23 items, e.g., “It is important for me to find my own path,” α = 0.70).

#### Satisfaction With Life

The Satisfaction with Life Scale (SWLS) was validated in Norway (Clench-Aas et al., 2011) and consists of five items (e.g., “In most ways, my life is close to my ideal,” 1 = totally disagree; 7 = totally agree). The mean sum scores ranged from 1 to 7. Reliability in the present study was α = 0.88.

#### Anxiety and Depression

Symptoms of anxiety (7 items, e.g., “I can suddenly get a feeling of panic”) and depression (7 items, e.g., “I feel like everything’s going slower”) were included as control variables in the regression models and were measured with the Hospital Anxiety and Depression Rating Scale (HADS) and validated in Norway (Bjelland et al., 2002). The ratings ranged from 0 (no problem) to 3 (maximum problem). Subscale scores (range: 0–21) were created by summing up the item scores. Reliability scores in the present study were α = 0.83 for anxiety and α = 0.71 for depression, respectively.

#### Resilience

Resilience was measured using the short version of the Connor–Davidson Resilience Scale (CD-RISC). The short version (CD-RISC2) (Vashnavi et al., 2007) consists of two items: “I am able to adapt when changes occur” and “I tend to bounce back after illness, injury, or other hardships” (0 = not true at all; 4 = true nearly all the time). The sum score ranged from 0 to 8 (Connor and Davidson, 2003). Reliability in the present study was α = 0.70.

### Analyses

In this explorative study, we investigated differences between people in late adulthood (≥65) and other adults (18–64) with independent sample *t*-tests for the continuous variables and chi-square tests for the categorical variables. In sub-analyses for the age group ≥ 75 years, one-way ANOVA were used for the continuous variables to compare means with the other age groups (18–64 and 65–74). Linear regression analyses were used to investigate how resilience and sources of meaning correlate with meaningfulness and satisfaction with life (Altman, 2018). We adjusted for demographic variables, anxiety, and depression. The standardized Beta (β) was reported. None of the variables included in the multivariate analysis showed collinearity over 0.70 (see Supplementary Tables 1, 2; Field, 2018). Linear regressions were also performed in sub-analyses for the age group ≥ 75 years. The significance level was set at *p* ≤ 0.05. The analyses were performed using SPSS version 27.

### Ethics

The Norwegian Regional Committees for Medical Research Ethics found that the present study was not covered by the Norwegian Health Research Act. It was therefore approved by the privacy representative at the Norwegian Centre for Research Data (NSD) (Project #42438) according to the Norwegian Personal Data Act. In advance of the study, the informants received information, including a notice that they would be providing their consent by submitting the questionnaire. The participants were given the opportunity to ask questions and could withdraw from the study at any time until publication of the study.

## Results

### Differences Between Old and Young Adults

Older adults reported significantly higher levels of meaningfulness compared to younger adults (3.5 vs. 3.3) (see Table 1). Correspondingly, significantly higher levels of the sources of meaning order and traditions (3.6 vs. 3.3), vertical self-transcendence (2.3 vs. 1.8), and horizontal self-transcendence (3.3 vs. 3.1) were measured. However, levels of accomplishment were significantly lower among older adults than among the younger ones (3.0 vs. 3.2). No differences in resilience, satisfaction with life, or the sources of meaning well-being and relatedness and liberality were observed between the groups. In the sub-analysis, when comparing the age group ≥ 75 with the other age groups, the score on meaningfulness was highest among the oldest (3.6 – not shown) and lower among the groups 65–74 and 18–64 (3.4 and 3.3, respectively – not shown). In the sub-analysis no significant differences between the age groups were found for resilience and satisfaction with life.

**TABLE 1:** Demographics and clinical characteristics of the sample divided into age groups.

	Total Sample (*N* = 925)	Young adults < 65 years (*N* = 706)	Old adults ≥ 65 years (*N* = 219)	*p*
Age (SD)	51.66 (16.57)	45.05 (12.76)	73.00 (6.25)	**<0.001**
*Sex*, *N* (*%*)				**0.020**
Female	504 (54)	400 (57)	104 (48)	
Male	421 (46)	306 (43)	115 (52)	
*Civil status*, *N* (*%*)				0.61
Pair-bonded	643 (70)	494 (71)	149 (69)	
Single	272 (30)	204 (29)	68 (31)	
*Education*, *N* (*%*)				**0.001**
>12 years	549 (60)	441 (63)	108 (50)	
≤12 years	372 (40	263 (37)	109 (50)	
*Meaningfulness* (*Range 0–5*), *mean* (*SD*)	3.3 (0.9)	3.3 (0.9)	3.5 (0.9)	**0.030**
*Sources of meaning* (*Range 0–5*), *mean* (*SD*)				
Well-being and relatedness	3.4 (0.6)	3.4 (0.6)	3.4 (0.7)	0.89
Order and traditions	3.3 (0.6)	3.3 (0.6)	3.6 (0.6)	**<0.001**
Vertical self-transcendence	1.9 (1.2)	1.8 (1.2)	2.3 (1.3)	**<0.001**
Horizontal self-transcendence	3.1 (0.7)	3.1 (0.6)	3.3 (0.7)	**0.002**
Accomplishment	3.1 (0.6)	3.2 (0.6)	3.0 (0.7)	**<0.001**
Liberality	3.0 (0.7)	3.0 (0.7)	2.9 (0.7)	0.46
*Satisfaction with life* (*Range 1–7*), *mean* (*SD*)	5.2 (1.2)	5.2 (1.2)	5.3 (1.2)	0.25
*Resilience* (*Range 0–8*), *mean* (*SD*)	6.7 (1.2)	6.8 (1.2)	6.6 (1.2)	0.15

*p ≤ 0.05, significant in bold.*

### Sources of Meaning and Resilience – Relations to Meaningfulness

Several of the sources of meaning were significantly and positively related to meaningfulness (see Table 2) in the multivariate regression analysis. The differences between those aged ≥ 65 and those aged 18–64 were small: well-being and relatedness (β0.21 vs.17), horizontal self-transcendence (β0.18 vs.16), accomplishment (β0.23 vs.19), and vertical self-transcendence (β0.47 vs. 46), with the latter having the strongest association. Liberality was negatively associated with meaningfulness in both age groups (β−0.12 vs. −0.13). No significant associations were found for order and traditions. Among the older group, men had lower levels of meaningfulness. In a sub-analysis of the oldest of the old (≥75, *N* = 71), it was found that vertical self-transcendence (β0.47, *p* < 0.001 – not shown) and accomplishment (β0.29, *p* = 0.02 – not shown) were the only sources of meaning positively associated with meaningfulness. Resilience was not associated with meaningfulness among the older adults (see Table 2). However, a significant positive association was found in the 18–64 age group (β0.07).

**TABLE 2:** Bivariate and multivariate linear regression of the associations between demographics, anxiety, depression, sources of meaning, resilience, and meaningfulness (dependent variable).

	Young and middle-aged adults				Old adults ≥ 65 years (*N* = 219)			
	< 65 years (*N* = 706)							
		
	Bivariate		Multivariate		Bivariate		Multivariate	
	β	*p*	β	*p*	β	*p*	β	*p*
Age	0.15	**<0.001**	0.07	**0.008**	0.03	**<0.001**	–0.08	**0.046**
Sex (women, men)	–0.20	**<0.001**	–0.05	0.075	–0.20	**0.004**	–0.10	**0.032**
Civil status (single, pair-bonded)	0.11	**0.003**	0.05	**0.047**	0.02	0.786	0.07	0.105
Education	0.07	0.068	0.02	0.421	0.09	0.207	0.03	0.519
**Sources of meaning**								
Well-being and relatedness	0.51	**<0.001**	0.17	**<0.001**	0.64	**<0.001**	0.21	**0.001**
Order and traditions	0.27	**<0.001**	0.05	0.110	0.47	**<0.001**	0.07	0.201
Vertical self-transcendence	0.55	**<0.001**	0.46	**<0.001**	0.66	**<0.001**	0.47	**<0.001**
Horizontal self-transcendence	0.49	**<0.001**	0.16	**<0.001**	0.64	**<0.001**	0.18	**0.002**
Accomplishment	0.37	**<0.001**	0.19	**<0.001**	0.55	**<0.001**	0.23	**0.001**
Liberality	0.11	**0.004**	–0.13	**<0.001**	0.38	**<0.001**	–0.12	**0.044**
Resilience	0.21	**<0.001**	0.07	**0.015**	0.19	**0.005**	0.04	0.424
Symptoms of anxiety	–0.09	**0.025**	0.02	0.421	–0.07	**<0.001**	–0.05	0.283
Symptoms of depression	–0.28	**<0.001**	–0.24	**<0.001**	–0.14	**0.040**	–0.01	0.812

*β = Standardized Beta. p ≤ 0.05, significant in bold. R^2^ young and middle-aged 0.59, R^2^ old adults 0.69.*

### Resilience and Sources of Meaning – Relations to Satisfaction With Life

Among those ≥65 years old, no associations were found between sources of meaning and satisfaction with life (see Table 3). However, in a sub-analysis of the oldest of the old (≥75, *N* = 71), it was found that accomplishment was positively associated with satisfaction with life (β0.32, *p* = 0.03 – not shown). For the 18–64 age group, a significant positive association with satisfaction with life was found for well-being and relatedness (β0.17) and a negative association for vertical self-transcendence (β-0.06). There was no significant association between resilience and satisfaction with life in the ≥65 age group. However, a positive, significant relationship was found in the 18–64 age group (β0.15; see Table 3).

**TABLE 3:** Bivariate and multivariate linear regression of the associations between demographics, anxiety, depression, sources of meaning, resilience, and satisfaction with life (dependent variable).

	Young and middle-aged adults				Old adults ≥ 65 years (*N* = 219)			
	< 65 years (*N* = 706)							
		
	Bivariate		Multivariate		Bivariate		Multivariate	
	β	*p*	β	*p*	β	*p*	β	*p*
***Demographic variables***								
Age	0.12	**0.002**	0.01	0.789	–0.05	0.470	0.05	0.405
Sex (women, men)	–0.11	**0.004**	–0.04	0.165	0.00	0.961	–0.15	**0.018**
Civil status (single, pair-bonded)	0.31	**<0.001**	0.21	**<0.001**	0.20	**0.003**	0.18	**0.002**
Education	0.13	**0.001**	0.04	0.208	0.19	**0.006**	0.05	0.453
Sources of meaning								
Well-being and relatedness	0.27	**<0.001**	0.17	**<0.001**	0.29	**<0.001**	0.07	0.457
Order and traditions	0.02	0.686	–0.01	0.836	0.12	0.074	–0.03	0.732
Vertical self-transcendence	–0.11	**0.003**	–0.06	**0.043**	–0.07	0.307	–0.09	0.146
Horizontal self-transcendence	0.10	**0.008**	0.01	0.812	0.19	**0.006**	0.07	0.366
Accomplishment	0.07	0.053	–0.01	0.791	0.25	**<0.001**	0.13	0.189
Liberality	–0.03	0.444	–0.03	0.373	0.17	**0.012**	–0.06	0.462
Resilience	0.42	**<0.001**	0.15	**<0.001**	0.39	**<0.001**	0.07	0.316
Symptoms of anxiety	–0.50	**<0.001**	–0.20	**<0.001**	–0.55	**<0.001**	–0.37	**<0.001**
Symptoms of depression	–0.60	**<0.001**	–0.33	**<0.001**	–0.49	**<0.001**	–0.21	**0.002**

*β = Standardized Beta. p ≤ 0.05, significant in bold. R^2^ Young and middle-aged 0.48, R^2^ Old adults 0.40.*

## Discussion

In the present study, the aim was to investigate sources of meaning and resilience, and the relations of these factors to meaningfulness and satisfaction with life. We sought to identify the special results among people in late adulthood (≥65 years) by comparing them with the rest of the population (18–64 years). This exploratory study aimed to generate knowledge in the Norwegian context as a preliminary knowledge base for possible policy and service development, considering health promotion for home-residing older people (≥65).

Regarding the first research question of whether there were differences between the age groups in terms of sources of meaning, resilience, meaningfulness, and satisfaction with life, the old age group (≥65) scored significantly higher on meaningfulness (see Table 1), though the effect size on this difference could be considered small (Cohen’s *d* = −0.17). In the sub-analysis of the age group ≥ 75 the score for meaningfulness was even higher, as opposed to studies reporting meaningfulness to decline in the older-old (Aftab et al., 2019; Steptoe and Fancourt, 2019). However, our finding is consistent with other research (Steger et al., 2006). Higher levels of meaningfulness among old people might be because this age group experience more fulfillment and existential stability in late life (Schnell, 2021). Similarly, the highly significant differences in the self-transcendence sources of meaning (see Table 1) could indicate that people in late adulthood more frequently search for objects beyond their immediate needs, with a vertical self-transcendence mean 1.8 vs. 2.3 (see Table 1), even though the effect size may not be considered large (Cohen’s *d* = −0.38).

In the present study, there were no differences between the age groups regarding satisfaction with life, despite that previous research has found satisfaction with life to increase in old age (Gana et al., 2013). It should be noted, however, that others found no correlation between satisfaction with life and age (Penick and Fallshore, 2005). In the present study there were no significant differences between the age groups in terms of resilience, even though lower scores on resilience for people in late adulthood have been found in previous research (Rothermund and Brandtstädter, 2003). This could be due to the mean age of 73 years, which could indicate a relatively young composition of participants in the age group ≥ 65 years in the present sample. However, nor the sub-analysis of the oldest of the old (≥75 – not shown) revealed differences neither in satisfaction with life nor resilience when comparing them with the other age groups.

The second research question asked about the association between sources of meaning and meaningfulness. Except for order and traditions, all sources of meaning were related to meaningfulness, and vertical self-transcendence and accomplishment had the strongest positive association (see Table 2). Conversely, liberality was negatively related. In a sub-analysis of the oldest of the old (≥75 – not shown), it was found that only vertical self-transcendence and accomplishment were associated with meaningfulness. Although there is some uncertainty regarding whether this sub-analysis was under powered because of the low number of cases (*N* = 71), it could indicate that these two sources are the most important when determining sources of meaning relevant for meaningfulness among people in late adulthood.

Vertical self-transcendence, which includes spirituality and religiousness, had the lowest score among the sources of meaning, although it was higher among the older participants than among the younger ones (see Table 1). This may reflect the expansion of secularity in the Norwegian context (Sørensen et al., 2012), which has been observed in other Scandinavian countries as well (Pedersen et al., 2018). At the same time, the older age group represents a life phase where a reversal of self-centering emerges, and a search for objects beyond one’s immediate needs seems to become more prominent (Schnell, 2021). Vertical self-transcendence was the strongest source of meaning related to meaningfulness in the multivariate analysis (see Table 2) where this variable alone represented 15.5% of the unique explained variance in meaningfulness after squaring the semi partial correlation (not shown). Thus, for individuals affiliated with religion and spirituality and the search for a supernatural reality, vertical self-transcendence may be highly important to their experience of meaning. Several resources, such as secular goals and beliefs, are available to everybody (Dezutter and Corveleyn, 2013). As such, people affiliated with a supernatural entity through beliefs and practices often have more resources available to them, as transcendent meaning systems appears to have a special ability to provide meaning, especially when no rational explanations of events are available (Pargament et al., 2005).

An important sub-scale of the accomplishment dimension is generativity, emphasizing an unselfish engagement for the community and future generations (Schnell, 2021). In previous studies, it has been shown that generativity has a strong association with meaningfulness (Schnell, 2011; Sørensen et al., 2019). Thus, it may be argued that selfless commitment to the good of the community and the next generations, as well as spiritual and religious approaches, may be the strongest sources of meaning for the old age group when related to meaningfulness. For those in the oldest age group, individual and self-centered awareness seemed to be less important in the material when seen together with meaningfulness, than it was for those in the lower age groups. According to Reker and Wong (2012), people find deeper meaning through commitment to a larger social cause and through values encompassing cosmic meaning and ultimate purpose, whereas the realization of personal potential and personal comfort may be less important for the meaning experience. This seems to be more obvious in old age if we follow gerotranscendence theory (Törnstam, 1989; 1997). Gerotranscendence theory describes a shift of meta-perspective, with more focus on internal values as well as the transcendent and the communal and less on materialistic and rational views. It is claimed that older adults optimize their performance related to selected and well-known values (Riediger et al., 2005). In the old age group vertical self-transcendence with religiousness and spirituality and accomplishment including generativity may represent such well-known and selected values. The finding that liberality was negatively associated with meaningfulness may also contribute to the understanding of gerotranscendence (Törnstam, 1989, 1997). Values such as freedom and individualism are highly valued in our modern Western society (Aakvaag, 2018). However, when seen in relation to meaningfulness, these values may appear less important for meaningfulness in old age, which also may be the case for other materialistic and individual views.

Previous research has found that hedonic sources like family and leisure were associate with satisfaction with life (Baumeister et al., 2013; Dewitte et al., 2021). However, the answer to our third research question is that none of the sources of meaning were related to satisfaction with life (see Table 3). Satisfaction with life is a construct representing a cognitive and global evaluation of life as a whole (Pavot and Diener, 2008), and is often understood as hedonic well-being (Keyes et al., 2002). It could be argued that the sources of meaning in SoMe are constructs especially related to meaningfulness, and thus they are characterized as eudaimonic and not related to satisfaction with life (Penick and Fallshore, 2005). At the same time, it is quite clear that sources in SoMe like well-being and relatedness (with sub-scales measuring enjoyment and sensitivity in private and with company) as well as liberality (with its sub-scales of freedom and comfort) would fall under what could be called hedonic sources (Baumeister et al., 2013). The positive association of the SoMe dimension well-being and relatedness with satisfaction with life in the 18–64 age group could substantiate this point (see Table 3). Still, there was no significant association between either eudaimonic or hedonic sources and satisfaction with life among people in late adulthood in the present sample. However, in the sub-sample of the oldest old (≥75), there was a positive association between the eudaimonic source accomplishment and satisfaction with life. Interestingly, this finding did not correspond with satisfaction with life, above described as hedonic well-being. Other measures of well-being, with a stronger focus on present or immediate satisfaction with life, could have a stronger relationship with the hedonic sources (Penick and Fallshore, 2005).

In the second and the third research questions, we asked if there was an association between resilience and meaningfulness, and between resilience and satisfaction with life, respectively. Based on partially coincident constructs between resilience and meaningfulness (Schnell, 2009, 2011; Bolton et al., 2016), and claims of an association between resilience and meaning (Reker and Wong, 2012), a positive association between resilience and meaningfulness could have been expected, even though direction between them would not be determined according to the research design. Also, positive associations between resilience and satisfaction with life was found in previous research (McCanlies et al., 2018; Jones et al., 2019). Interestingly, associations were not found in the present study neither between resilience and meaningfulness, nor between resilience and satisfaction with life.

Whereas resilience is often investigated in selected samples including participants who are struggling with demanding life events (McCanlies et al., 2018; Jones et al., 2019), the present sample was population based. Resilience was measured with the CD-RISC2, which included only two items (Vashnavi et al., 2007) and thus may have provided limited information about resilience, failing to capture the variety of domains pertaining to this concept (Infurna and Luthar, 2017). However, given that positive associations were found between resilience and meaningfulness as well as between resilience and satisfaction with life in the younger age group (see Tables 2, 3), there could be other explanations for the lack of significant associations between these domains among old adults (see Tables 2, 3).

It has been argued that younger people may struggle with demands in society and rely on resources like resilience and emotional strength, such as the ability to adapt and reintegrate despite experiencing adversity (Herman et al., 2011). Even if people in old age are retired and do not struggle with the demands of career and work requirements, they increasingly face demanding life situations, such as various losses, disabilities, and health challenges (Eurostat, 2020). While no association was seen between resilience and satisfaction with life, nor between resilience and meaningfulness among the older participants in the present study (see Tables 2, 3), the shift of meta-perspective in late adulthood from striving for a career and other individualistic and materialistic approaches to communal and transcendent entities (Törnstam, 1989, 1997) could contribute to an interpretation of this finding. The older participants in the present study may lean on the satisfaction with life that their life phase entails—a phase where fulfilment and existential stability can be expected to a great extent (Schnell, 2021). Since the sample in this explorative study contained only individuals with a permanent address connected to a flat or house, we argue that the material is context bound to home-dwelling people and reflects their view. It could be that resilience in terms of elasticity is not triggered in this group since they represent people in stable, safe conditions and with a good socio-economic background (Colerick, 1985). It should also be noted that Norway has the highest rank in the world in terms of material living conditions and satisfaction with life (The Organisation for Economic Co-operation and Development, 2020).

### Strengths and Limitations

The strengths of the present study are the big sample size and random selection from the Norwegian National Registry. An obvious limitation of the study was its cross-sectional design, with no possibility of showing causal relationships. Another limitation was the response rate. Even though the 7,500 invited were randomly selected, it can be questioned whether the final sample can be designated as population based due to the 12.2% response rate. More effort could have been made to reach non-responders, for instance, using the Total Design Method (Dillman, 1978), but the recruitment procedure described above was approved according to Norwegian ethical standards.

The sample was skewed in terms of education level (60% in the sample vs. 30% in the population had >12 years of education) (Statistics Norway, 2021), which could have an impact on the results. At the same time, the frequency of church attendance measured in the sample (not shown) was similar to that in other population-based studies in Norway (Sørensen et al., 2012), which may indicate that the present investigation is not biased by overrepresentation of participants interested in religion, spirituality, and other transcendent approaches. Demographic variables may have relevance to the concepts in the research questions. The distributions of age (mean 51.7 years vs. 47.7 among the invited) and gender (54.5% women vs. 49.4% among the invited) in the final sample could be considered acceptable compared to the general population. Moreover, the share of people living alone (30.0%) was close to that in the general population (25.6%) (Norwegian Institute of Public Health, 2020). Against this background, it could be argued that the sample is representative of the Norwegian population.

The sub-analysis of the age group ≥ 75 years was performed knowing that the sample size (*N* = 71) was potentially too small for the number of variables included in the regression model. Accordingly, there are clear reservations when illustrating and elaborating the findings for the total sample of the old age group (≥65 years).

It is possible that constructs overlapped in the analyses. However, all Pearson correlation coefficients were lower than 0.70 (Field, 2018) when testing for collinearity between the employed continuous variables (see Supplementary Tables 1, 2). Resilience was measured with a two-item instrument (CD-RISK2), which may not have grasped all the domains relevant for this concept.

## Conclusion

In the present Norwegian population-based study, it was found that people in late adulthood (≥65 years) scored significantly higher on meaningfulness compared to younger adults (age 18–64). Of the sources of meaning, vertical self-transcendence, including explicit religiosity and spirituality, had the strongest relation to meaningfulness for people in late adulthood, after adjusting for demographics, anxiety, and depression. For the same group, accomplishment, including generativity and unselfish engagement with the surroundings and future generations, also stood out as a prominent source of meaning when related to meaningfulness. No sources of meaning were associated with satisfaction with life in the older group. No associations between resilience and meaningfulness, nor between resilience and satisfaction with life, were found among people in late adulthood. However, positive associations were found between resilience and meaningfulness, as well as between resilience and satisfaction with life, in the 18–64 age group.

## Implications

The present study was cross-sectional and exploratory. Further investigations are needed employing longitudinal research designs to examine whether eudaimonic domains like vertical self-transcendence and accomplishment impact meaningfulness among older adults. Intervention studies with selected samples may also be relevant. Such studies could investigate the significance of approaches connected to the most relevant sources of meaning, like those shown in the present study. If it is true that vertical self-transcendence and accomplishment are the most prominent sources of meaningfulness, this would strengthen the theory of gerotranscendence and the understanding of older people’s emphasis on communal and transcendent views in the Norwegian context.

We sought to search for sources associated with meaningfulness in old age. Such knowledge may facilitate health-promoting approaches for this age group (Knitzek et al., 2021). Meaning in life is a personal, subjective, and private issue (Reker and Wong, 2012). Health-promoting approaches emphasizing meaningfulness and its sources should therefore consider individuals needs and preferences (Slettebø et al., 2017). It has been suggested to support older people in continuing their discovery and creation of meaning by focusing on the different sources mentioned in the present article, for example, using life review (life history) as a clinical technique in settings with counselors (Penick and Fallshore, 2005). Another suggested intervention might be writing courses for older adults living at home (Lehmann and Brinkmann, 2019). As it is crucial in the communal view of older people (Törnstam, 1989) to perceive reciprocity in relationships and a sense of still having the ability to influence their communities (Bahl, 2018), creative writing and storytelling in groups have been proposed as aesthetic and existential practices in community with others (Synnes, 2015). Enabling customized religious and spiritual practices for older people who are not able to come to religious services on their own could be a more practically oriented approach. Regarding self-transcendence and the search for objects beyond immediate needs, the activities mentioned above may contribute to a deeper sense of meaningfulness. As meaningfulness may contribute to successful aging, resulting in better health and longevity (Steptoe and Fancourt, 2019), these approaches, among others, could help older people to stay at home longer (Norwegian Government, 2017).

## Data Availability Statement

The datasets presented in this article are not readily available because participants did not give their approval for sharing data. Requests to access the datasets should be directed to TS, torgeir.sorensen@vid.no.

## Ethics Statement

The study involved human participants and was reviewed and approved by the Norwegian Centre for Research Data (NSD) (Project #42438). The participants provided their written informed consent to participate in this study.

## Author Contributions

TS collected the data and wrote the first draft of the manuscript. TS, KH, and EG contributed to the conception and design of the study, analyzed the data, contributed to critical reading, manuscript revision, final manuscript preparation, and approved the submitted version.

## Conflict of Interest

The authors declare that the research was conducted in the absence of any commercial or financial relationships that could be construed as a potential conflict of interest.

## Publisher’s Note

All claims expressed in this article are solely those of the authors and do not necessarily represent those of their affiliated organizations, or those of the publisher, the editors and the reviewers. Any product that may be evaluated in this article, or claim that may be made by its manufacturer, is not guaranteed or endorsed by the publisher.
