# Supplementary material for: Relationships of Sources of Meaning and Resilience With Meaningfulness and Satisfaction With Life: A Population-Based Study of Norwegians in Late Adulthood
Source: Front Psychol. 2021 Dec 2;12:685125. doi: 10.3389/fpsyg.2021.685125 (PMC8674485; doi:10.3389/fpsyg.2021.685125)
Supplement: Supplementary file 1 [file Data_Sheet_1.docx]

**Supplemental – Syntax for regression analysis – Table 2 and 3**

DATASET ACTIVATE DataSet1.

SORT CASES BY Age_dik_65.

SPLIT FILE LAYERED BY Age_dik_65.

REGRESSION

/MISSING LISTWISE

/STATISTICS COEFF OUTS R ANOVA

/CRITERIA=PIN(.05) POUT(.10)

/NOORIGIN

/DEPENDENT Meaningfulness (Table 2) or Satisfaction with life (Table 3)

/METHOD=ENTER age sex Family_dik1 education WB_R Order Vertical_ST Horizontal_ST ACC LIB RECIL_T

HADS_T_anxi HADS_T_depr.
