# Supplementary material for: Relationships of Sources of Meaning and Resilience With Meaningfulness and Satisfaction With Life: A Population-Based Study of Norwegians in Late Adulthood
Source: Front Psychol. 2021 Dec 2;12:685125. doi: 10.3389/fpsyg.2021.685125 (PMC8674485; doi:10.3389/fpsyg.2021.685125)
Supplement: Supplementary file 2 [file Table_1.docx]

**Supplemental – Table 1. Bivariate Pearson correlations between the main variables, age group 18-64 years (N=706).**

|  | 1. | 2. | 3. | 4. | 5. | 6. | 7. | 8. | 9. | 10. | 11. |
| --- | --- | --- | --- | --- | --- | --- | --- | --- | --- | --- | --- |
| Meaningfulness (1) | 1 |  |  |  |  |  |  |  |  |  |  |
| Well-being and relatedness (2) | .51** | 1 |  |  |  |  |  |  |  |  |  |
| Order and traditions (3) | .27** | .37** | 1 |  |  |  |  |  |  |  |  |
| Vertical self-transcendence (4) | .55** | .28** | .25** | 1 |  |  |  |  |  |  |  |
| Horizontal self-transcendence (5) | .49** | .46** | .17** | .29** | 1 |  |  |  |  |  |  |
| Accomplishment (6) | .37** | .38** | .15** | .16** | .46** | 1 |  |  |  |  |  |
| Liberality (7) | .11** | .32** | .15** | .04** | .30** | .59** | 1 |  |  |  |  |
| Satisfaction with Life (8) | .30** | .27** | .02 | -.11** | .10** | .07 | -.03 | 1 |  |  |  |
| Resilience (9) | .21** | .13** | .03 | -.09* | .10** | .25** | .08* | .42** | 1 |  |  |
| Symptoms of anxiety (10) | -.09* | -.05 | .05 | .13** | .10* | .11** | .08* | -.50** | -.37** | 1 |  |
| Symptoms of depression (11) | -.28** | -.26** | .03 | .17** | -.10** | -.11** | .01 | -.61** | -.45** | .60** | 1 |

**Note**: 2-6 = Sources of Meaning. ** p ≤ .01; * p ≤ .05.
