# Supplementary material for: Relationships of Sources of Meaning and Resilience With Meaningfulness and Satisfaction With Life: A Population-Based Study of Norwegians in Late Adulthood
Source: Front Psychol. 2021 Dec 2;12:685125. doi: 10.3389/fpsyg.2021.685125 (PMC8674485; doi:10.3389/fpsyg.2021.685125)
Supplement: Supplementary file 3 [file Table_2.docx]

**Supplemental – Table 2. Bivariate Pearson correlations between the main variables, age group ≥ 65 years (N=219).**

|  | 1. | 2. | 3. | 4. | 5. | 6. | 7. | 8. | 9. | 10. | 11. |
| --- | --- | --- | --- | --- | --- | --- | --- | --- | --- | --- | --- |
| Meaningfulness (1) | 1 |  |  |  |  |  |  |  |  |  |  |
| Well-being and relatedness (2) | .64** | 1 |  |  |  |  |  |  |  |  |  |
| Order and traditions (3) | .47** | .66** | 1 |  |  |  |  |  |  |  |  |
| Vertical self-transcendence (4) | .66** | .36** | .34** | 1 |  |  |  |  |  |  |  |
| Horizontal self-transcendence (5) | .63** | .58** | .45** | .44** | 1 |  |  |  |  |  |  |
| Accomplishment (6) | .55** | .57** | .34** | .28** | .65** | 1 |  |  |  |  |  |
| Liberality (7) | .38** | .57** | .41** | .22** | .50** | .71** | 1 |  |  |  |  |
| Satisfaction with Life (8) | .22** | .29** | .12 | -.07 | .19** | .25** | .17* | 1 |  |  |  |
| Resilience (9) | .19** | .24** | .08 | -.06 | .15* | .27** | .20** | .39** | 1 |  |  |
| Symptoms of anxiety (10) | -.07 | -.18** | -.07 | .14* | -.06 | -.14* | -.15* | -.55** | -.48** | 1 |  |
| Symptoms of depression (11) | -.14* | -.23** | .01 | .05 | -.11 | -.18** | -.12 | -.49** | -.47** | .57** | 1 |

**Note**: 2-6 = Sources of Meaning. ** p ≤ .01; * p ≤ .05.
